# European Journal of Psychiatric Trainees - a new scientific peer-reviewed Journal in Psychiatry

**DOI:** 10.1192/j.eurpsy.2023.2380

**Published:** 2023-07-19

**Authors:** F. Santos Martins, M. J. Santos, L. Afonso Fernandes, D. Cavaleri, M. Pinto da Costa, N. Žaja, K. Markin, L. Tomašić, H. Ryland, J. D King, L. E Stirland, A. Seker

**Affiliations:** 1 Psychiatry Department, Centro Hospitalar Universitário S. João; 2 CINTESIS, Faculty of Medicine, University of Porto, Porto; 3 Department of Mental Health, Hospital Prof. Doutor Fernando Fonseca, Amadora, Portugal; 4 Department of Medicine and Surgery, University of Milano-Bicocca, Monza, Italy; 5 Institute of Biomedical Sciences Abel Salazar, University of Porto, Porto, Portugal; 6 South London and Maudsley NHS Foundation Trust; 7 Institute of Psychiatry, Psychology and Neuroscience, King’s College London, London, United Kingdom; 8 University Psychiatric Hospital Vrapče, Zagreb, Croatia; 9 Department of Psychiatry, Military Medical Academy, St. Petersburg, Russian Federation; 10 Department of Psychiatry, University of Oxford, Oxford; 11 Division of Psychiatry, Imperial College London, London, United Kingdom; 12 Global Brain Health Institute, University of California, San Francisco, United States

## Abstract

**Introduction:**

Psychiatry training programs vary in the degree to which they offer trainees with an opportunity to get involved in research. Exposure to research during the training period is critical, as this is usually when trainees start their own scientific research projects and gain their first experiences in academic publishing.

**Objectives:**

We present the European Journal of Psychiatric Trainees (EJPT) (ejpt.scholasticahq.com), the official journal of the European Federation of Psychiatric Trainees (EFPT), including its scope, mission and vision and practical considerations.

**Methods:**

Reflecting on the foundation and operation of the European Journal of Psychiatric Trainees.

**Results:**

The European Journal of Psychiatric Trainees is an Open Access, double blind peer-reviewed journal which aims to publish original and innovative research as well as clinical, theory, perspective and policy articles, and reviews in the field of psychiatric training, psychiatry and mental health. Its mission is to encourage research on psychiatric training and inspire scientific engagement by psychiatric trainees. Work conducted by psychiatric trainees and studies of training in psychiatry are prioritized. The journal is open to submissions, and while articles from psychiatric trainees are prioritized, submissions within scope from others are also encouraged. The article processing fee is very low and waivable. It is planned to publish two issues yearly.

The first article was published in July 2022, titled “Fluoxetine misuse by snorting in a teenager: a case report” and it received 218 views as of 17 October 2022, which confirms the journal’s potential for visibility.

**Conclusions:**

The European Journal of Psychiatric Trainees is a non-profit initiative designed to offer psychiatric trainees a platform to publish and gain experience in publishing. Thanks to its robust double blind peer reviewing system, it has the potential to contribute to scientific excellence.

**Disclosure of Interest:**

None Declared

